# Late Holocene cooling drove drastic decreases in cladoceran diversity in a subarctic lake

**DOI:** 10.1038/s41598-024-81690-7

**Published:** 2024-12-16

**Authors:** María de los Ángeles González Sagrario, Tobias Vrede, Simon Belle

**Affiliations:** 1 Instituto de Investigaciones Marinas y Costeras (IIMYC), Universidad Nacional de Mar del Plata, CONICET, J. B. Justo 2550, 7600 Mar del Plata, Argentina; 2 Department of Aquatic Sciences and Assessment, Swedish University of Agricultural Sciences, Box 7050, 750 07 Uppsala, Sweden

**Keywords:** Biodiversity, Climate change, Holocene, Terrestrial-aquatic linkage, Zooplankton, Zoobenthos, Climate change, Limnology, Palaeoecology

## Abstract

Subarctic lakes are sentinels of climate change, showing responses in their physical, chemical, and biological properties. However, climate-induced changes in invertebrate diversity and their underlying mechanisms are not fully understood. We explored the relationship between past climate change and taxonomic composition of subfossil cladocerans in a subarctic lake during the last *ca.* 5700 years. The Cladocera community shifted from specialist to generalist species at *ca.* 3500 cal years BP, corresponding to the long-term cooling period between the Holocene Thermal Maximum and the Late Holocene. Taxonomic diversity declined driven by the collapse of the keystone herbivorous *Daphnia longispina* group, pelagic and littoral predators, and phytophilous benthic species, therefore resulting in a simplification of the food web and a reduction of trophic levels. Furthermore, the shift in cladoceran composition was associated with the decline of aquatic primary producers and the development of birch forest, suggesting a potential causal link between dissolved organic carbon dynamics and cladoceran community composition. This study provides empirical evidence of the response of cladocerans to climatic fluctuations and their underlying mechanisms through catchment-mediated processes and direct temperature-induced changes.

## Introduction

Subarctic lakes have been recognized as sentinels of climate change as their physical, chemical, and biological properties can be directly and indirectly affected by climate change^1,2^. Among others, the direct effects of warming on lakes affect the timing and duration of ice cover^3^, and community composition^4^. Climate-driven landscape transformations (through vegetation changes in composition and productivity) can change run-off patterns, biogeochemical cycles, and organic matter dynamics^5^. Furthermore, climate change is currently affecting arctic and subarctic ecosystems at higher rates than elsewhere^1,6^. However, our understanding of how these ecosystems respond to climate change is limited by the lack of monitoring data. Learning from past changes using paleolimnological reconstructions offers a promising approach to addressing this knowledge gap.

In subarctic parts of Fennoscandia, the long-term Holocene climate change has profoundly transformed the landscape, through changes in vegetation composition and biomass^7,8^, and lake productivity^7,9–11^; thus, providing an exceptional natural set-up to investigate the complex links between climate fluctuations and aquatic biodiversity. After the last deglaciation, the landscape rapidly shifted from vegetation-free to forest during the early Holocene (*ca*. 9500–6500 cal years BP)^8,12^, and the warm and dry Holocene Thermal Maximum (*ca*. 6500–3500 cal years BP) allowed for the expansion of single pine (*Pinus sylvestris* L.). During the late Holocene (*ca*. 3500–500 cal years BP), mountain birch (*Betula pubescens* subsp. *czerepanovii* (N. I. Orlova) Hämet-Ahti) established in the area as a response to the long-term marked cooling and wetter conditions^7,8^. Changes in precipitation regimes and vegetation composition and biomass have key influences on the flux of dissolved organic matter (DOC) and nutrients to lakes^5^, affecting light availability for aquatic primary producers^13^, food quality for zooplankton^14^, and altering visual detection of zooplankton by fish^15^. Whereas the direct effects of Holocene climate change on subarctic lakes have been extensively studied^11,16–19^, the influence of climate-driven landscape modifications on aquatic diversity has been largely overlooked (but see^13^).

Cladocerans are key organisms in the pelagic and benthic food webs of lakes, transferring energy and nutrients from primary producers to higher trophic levels. Furthermore, cladocerans are one of the best-represented groups of invertebrates that leave identifiable remains in lake sediments and can thus be used as a reliable surrogate in biodiversity studies. Directional changes in cladoceran or other communities reflect species turnover, i.e., the gain, loss, or overlap of species along a spatial, environmental, or temporal gradient^20^. Most ecosystems are influenced by a few controlling variables that strongly influence the community structure and ecosystem functions^21^. Temperature is a key controlling variable in arctic and subarctic aquatic ecosystems for crustacean plankton^22^. In colder regions, the spatio-temporal distribution of cladocerans is regulated by regional climate, and the summer temperature is the main variable explaining species distribution^23,24^ and richness^22^. Other environmental factors such as food availability and selective predation of fish could also influence species and clone sorting, as well as changes in community size structure^25–27^. Paleolimnological studies based on cladoceran remains have shown shifts in community composition related to climate change and lake water levels in arctic/subarctic lakes^18,28,29^. Investigations in Finland, illustrate that Cladocera composition changed substantially during the Holocene following climate change^19,24^. However, the shift from mid to late Holocene differed among lakes, in some cases littoral cladocerans became dominant whereas in others pelagic taxa did^18,19,30^. The directional changes described imply an ecological reorganization, i.e., a shift in the community structure, affecting taxonomic diversity^31^. However, the effects on other dimensions of diversity such as horizontal and/or vertical diversity have not been explored. Horizontal diversity constitutes the taxonomic or functional richness and evenness of different entities (species, genes, etc.) within a single trophic level; thus, higher horizontal diversity promotes ecosystem stability^32^. Vertical diversity refers to the length of the food chain or the vertical niche breadth and has a destabilizing effect^32,33^. Conserving horizontal diversity across multiple trophic levels ensures ecosystem functionality and stability^33^. The cladoceran community in arctic/subarctic lakes includes primary consumers and pelagic and littoral predators, allowing for exploration of the impact of climate change on community structure and horizontal diversity at several trophic levels.

**Fig. 1:**
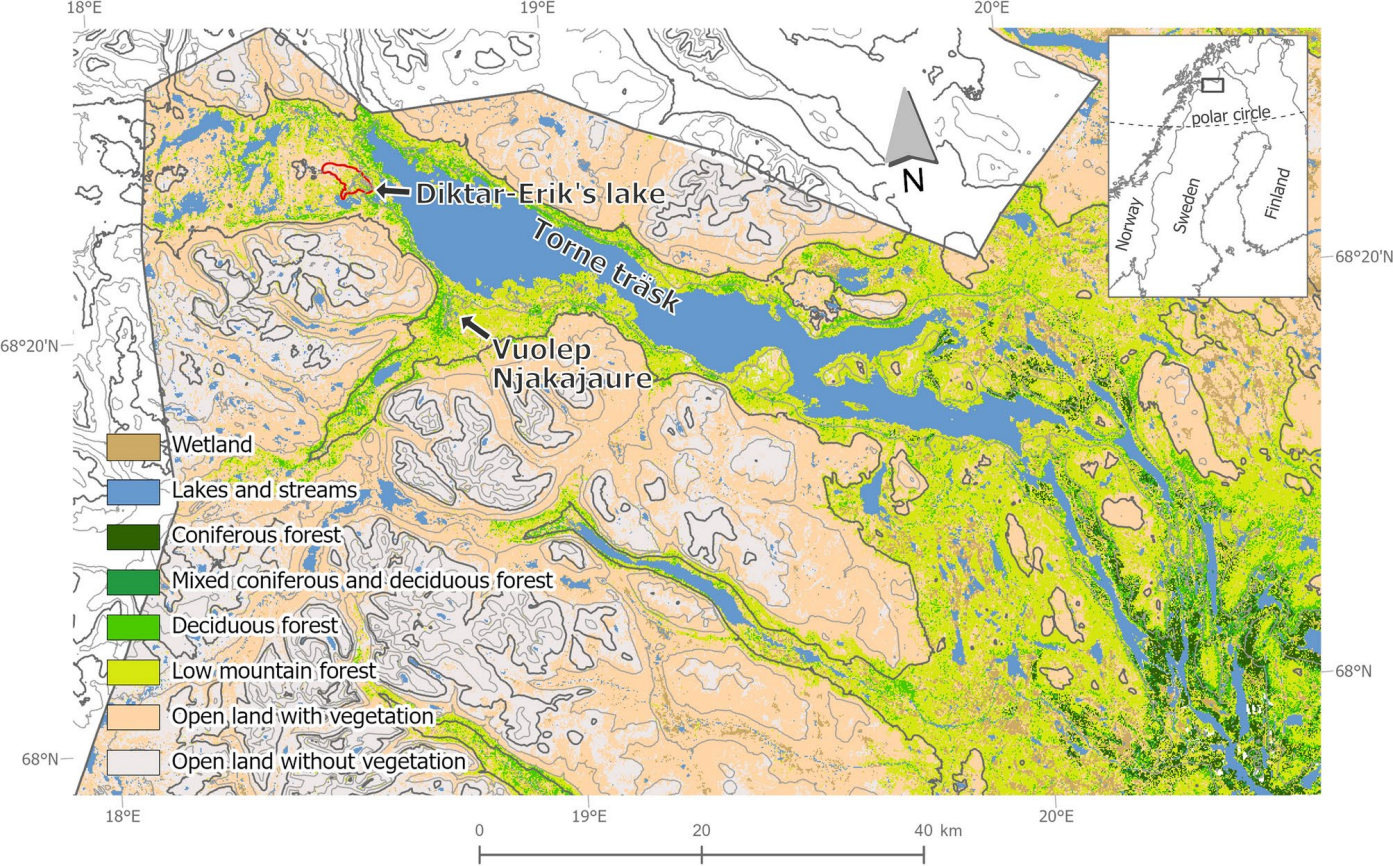
Land cover map of the Abisko region, Sweden, showing Diktar-Erik’ lake and its catchment (red line). The elevation isolines have 200 m equidistance, with the elevations 600 m.a.s.l. and 1200 m.a.s.l. being shown with thicker lines. Land cover data: Naturvårdsverket (Swedish Environmental Protection Agency), Swedish national land cover data 2018^52^, provided under a CC0 license. The resolution of the underlying data is 10 × 10 m pixels. Data were aggregated to 100 × 100 m pixels and forest vegetation classes were combined to enhance the visibility of general vegetation patterns across the landscape. Elevation data: GSD-Höjddata, grid 2 + © Lantmäteriet (the Swedish Land Survey). The map was created with ArcGIS pro v.3.2.1 (https://www.esri.com/en-us/arcgis/products/arcgis-pro/) and lake names and arrows were added later using Affinity Designer v.1.10.8.

Considering the profound changes that subarctic and arctic ecosystems are experiencing, it remains a challenge to understand the mechanisms involved in the variation and control of ecological responses, the direction of change in the cladoceran community under different climate fluctuations, and how the different dimensions of diversity are affected. To understand some of these knowledge gaps, several recent studies have recommended increasing monitoring programs of invertebrate abundance and distribution and combining environmental data with paleolimnological reconstructions to improve the understanding of mechanistic controls on invertebrate communities across spatio-temporal scales^1^. The main objective of this study was to unravel the long-term relationship between taxonomic composition of cladocerans and climate change in a subarctic lake by disentangling the respective influence of aquatic and terrestrial primary production. To do so, we analyzed Cladocera remains in the sediment record of Diktar-Erik´s lake (hereafter Diktar-Erik, Fig. 1) covering the last *ca*. 5700 cal years BP. We hypothesized that: (1) long-term cooling induced a loss of taxonomic and horizontal diversity, and (2) the shift in cladoceran community coincided with a decrease in primary production in the lake and changes in vegetation cover in the catchment occurring during the transition from Mid to the Late Holocene.

## Results

### Cladocera community composition and diversity patterns

**Fig. 2:**
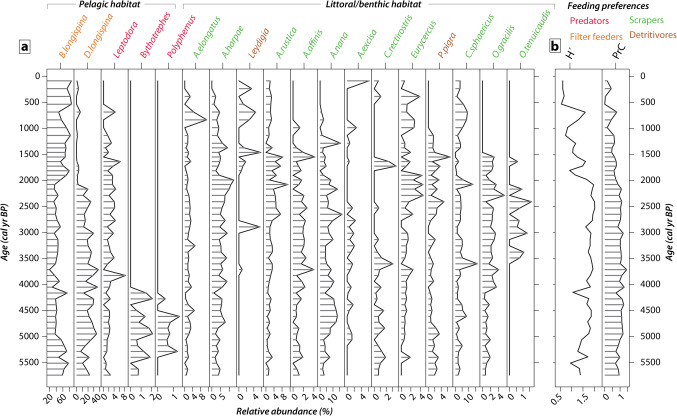
(**a**) Temporal trend in the taxonomic composition of Cladocera community in the sediment core from Diktar-Erik. Taxa contributions are expressed as relative abundance (%). Species names were abbreviated and full names are from left to right: *Bosmina longispina* Leydig, 1860, *Daphnia longispina* group (O.F. Müller, 1776), *Leptodora kindtii* (Focke, 1844)*, Bythotrephes longimanus* Leydig, 1860*, Polyphemus pediculus* (Linnaeus, 1758)*, Acroperus elongatus* (G.O. Sars, 1862), *Acroperus harpae* (W. Baird, 1834), *Leydigia* spp. W. Kurz, 1875, *Alona rustica* T. Scott, 1895, *Alona affinis* (Leydig, 1860), *Alonella nana* (W. Baird, 1843), *Alonella excisa* (S. Fischer, 1854), *Camptocercus rectirostris* Schoedler, 1862, *Eurycercus* spp. W. Baird, 1843, *Paralona pigra* (G.O. Sars, 1862), *Chydorus sphaericus* group (O. F. Müller, 1776), *Ophryoxus gracilis* (G.O. Sars, 1862), and *Oxyurella tenuicaudis* (G.O. Sars, 1862). For species allocation to habitats or trophic guilds see Supplementary, Table S1 online. (**b**) Shannon Diversity index (H’) and temporal trend of scores extracted from Principal Curve analysis (PrC).

A minimum number of 200 remains counted per sample, thus corresponding to a total of 16,966 remains in the 50 sediment layers, allowed to identify 18 predominant cladoceran taxa (Fig. 2a).

**Fig. 3:**
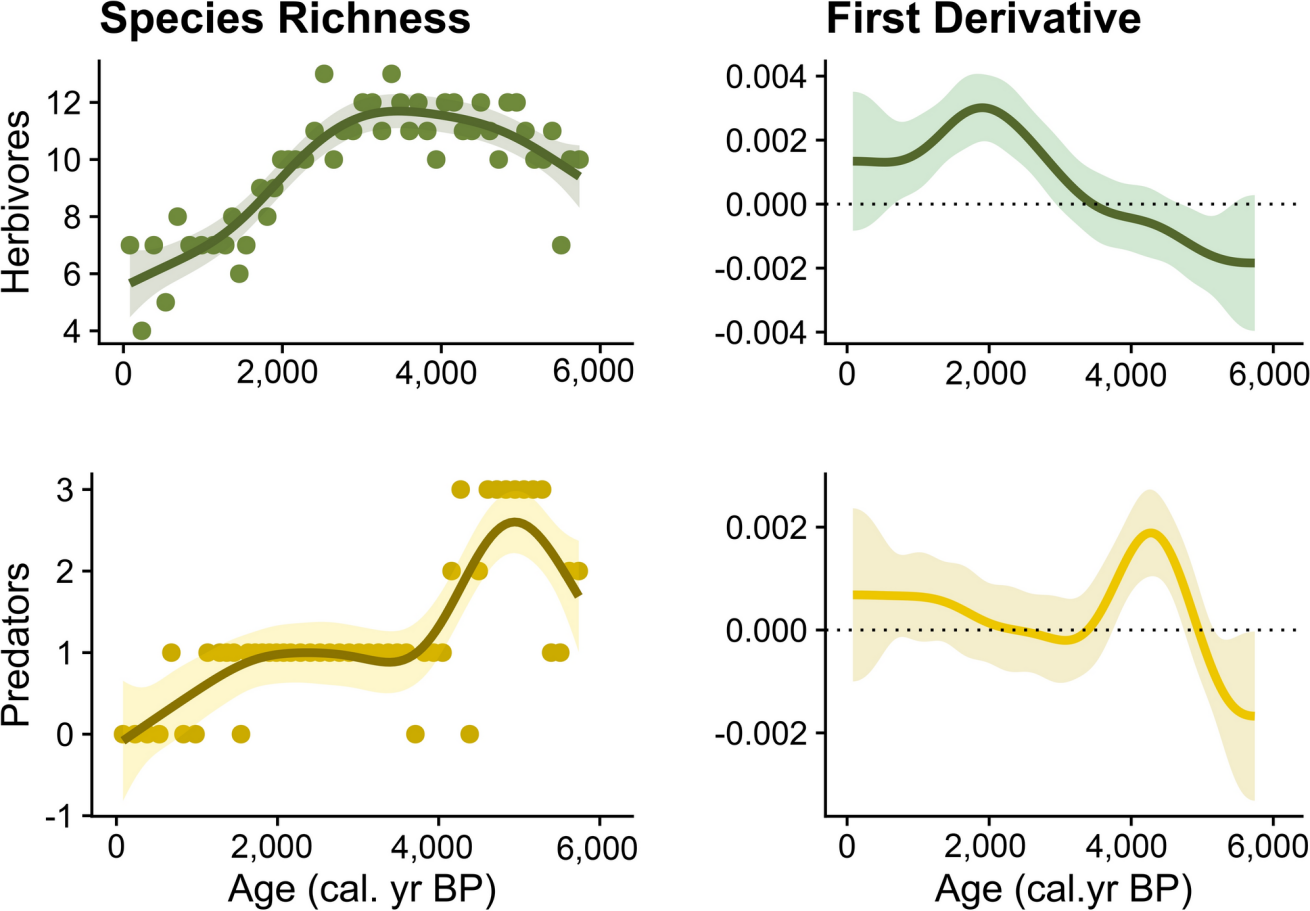
Horizontal diversity at two different trophic levels, primary (herbivores) and secondary consumers (pelagic predators). Graphs on the left show the GAM trend with a 95% confidence interval fitted to the temporal series of species richness. Graphs on the right show the first derivative of the fitted GAM trend and its confidence interval. The period of transition corresponds to those time points when the trend and its interval are away from zero.

The Cladoceran community changed in terms of species relative contribution and dominance/codominance, which is reflected by changes in Shannon’s diversity (H’), and changes in community composition (Principal Curves) over time (Fig. 2a, b). The GAM fitted to the temporal series of the Shannon diversity index explains 67.7% of the variation (e*df* of smooth term = 4.05, *P* value < 0.0001), and the first derivative identifies two periods (Supplementary, Fig. S3 online). From *ca*. 5700 to 2400 cal years BP, Shannon’s diversity index shows an increasing trend (Fig. 2b; Supplementary, Fig. S3 online). Initially, pelagic taxa dominated the community, composed of herbivores (*Bosmina longispina* and *Daphnia longispina* group) and predators (*Leptodora kindtii* and *Bythotrephes longimanus*), and littoral/benthic species contributed with low abundances. This community gradually shifted to a more even composition consisting of pelagic herbivores (*Bosmina longispina* and *Daphnia longispina*), a pelagic predator (*Leptodora kindtii*), and several littoral/benthic species with affinity to distinct substrates such as vegetated areas (e.g., *Alonella nana*) and sandy bottom (e.g., *Ophryoxus gracilis*, *Acroperus harpae*) (Fig. 2a). Despite this increase in taxonomic diversity, the number of species remains relatively stable (12–16 species). Starting around *ca*. 2400 cal years BP there is a reduction in the number of species from 13 to 7. This is reflected in the decline in Shannon’s diversity index due to the disappearance of common (e.g., *Daphnia longispina* group and *Alonella nana*) and less abundant taxa (e.g.,* Paralona pigra, Ophryoxus gracilis*, and *Oxyurella tenuicaudis*) (Fig. 2a, b; Supplementary, Fig. S3 online). In addition to taxonomic diversity, horizontal diversity also decreases over time, as shown by GAMs fitted to the species richness of primary (herbivores) and secondary (predators) consumers (Fig. 3). Both models account for a large percentage of the variation in species richness. For herbivores, the model accounts for 80.4% of the variation (*edf* of smooth term = 4.5, *P* value < 0.0001), and for predators, it accounts for 68.2% of the variation (*edf* of smooth term = 5.88, *P* value < 0.0001; Fig. 3). A decrease in herbivore species richness occurred around 2800 cal years BP during the transition to the Late Holocene. Predator diversity peaked during the Holocene Thermal Maximum and then declined around 4000 cal years BP, collapsing in the system by 500 cal years BP (Fig. 3). The pelagic community shifted from the codominance of *Daphnia longispina* group and *Bosmina longispina* to the dominance of the latter and the loss of predators (*Leptodora kindtii, Bythotrephes longimanus*). In the littoral-benthic community, species loss and replacement occurred. The littoral predator *Polyphemus pediculus* collapsed and the community shifted from phytophilous to generalist and pioneer species associated with different substrata like *Chydorus sphaericus* and *Acroperus elongatus* (Fig. 2a).

**Fig. 4:**
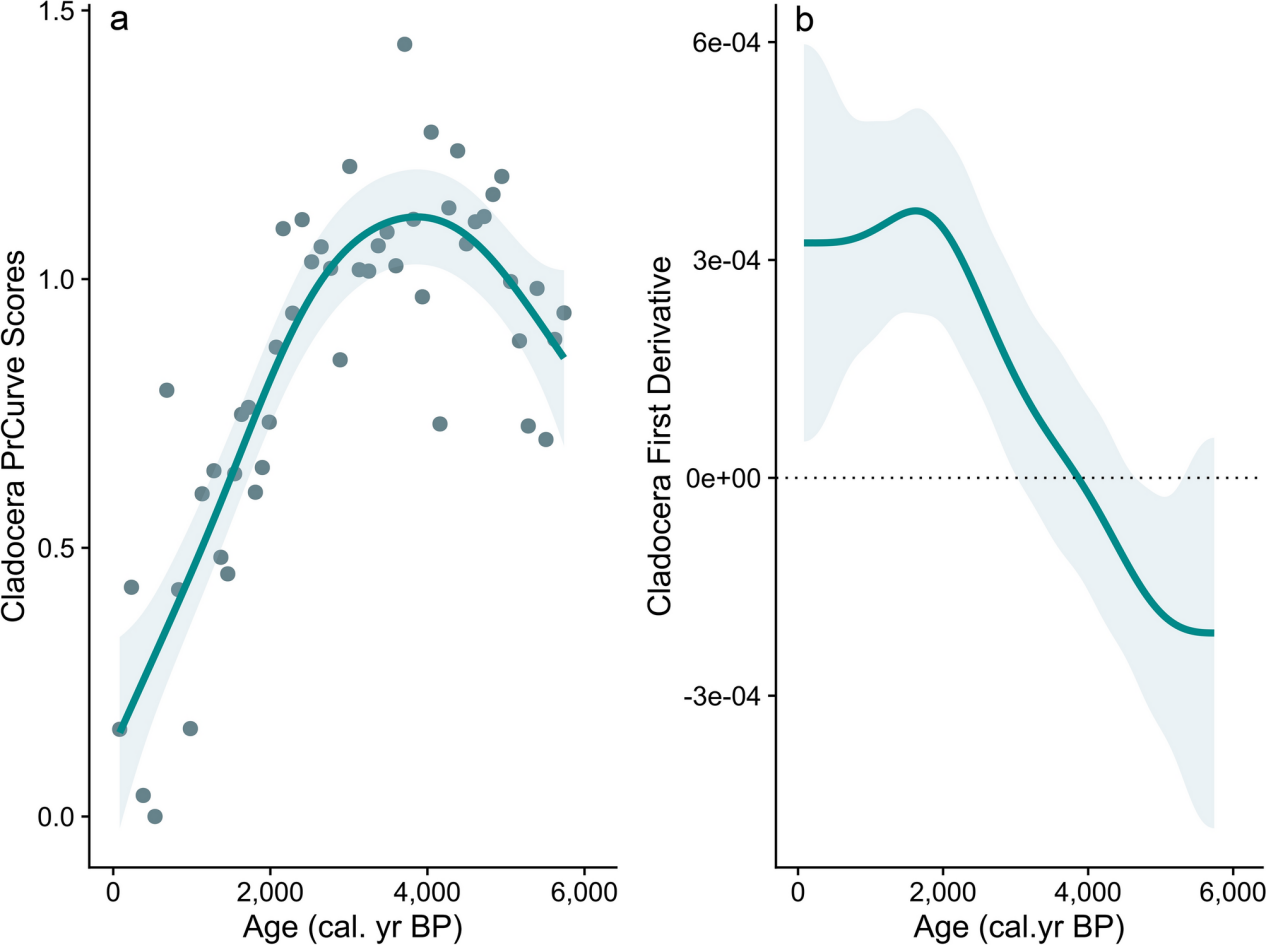
(**a**) Scores from Principal Curve (PrC) analysis of the cladoceran community (dots), and GAM trend with a 95% confidence interval fitted to the temporal series of PrC scores. (**b**) The first derivative of the fitted GAM trend and its confidence interval show the period of transition when both bounded away from the zero.

The Principal Curve analysis captured changes in species composition, reflecting 54% of the variation in the cladoceran community. The scores decline after *ca*. 3000 cal years BP (Fig. 2b), reflecting the change in species composition. The GAM fitted to the time series of the scores of the Principal Curve explained 76.4% of the data deviance (*edf* smooth term = 3.73, *P* value < 0.0001; Fig. 4a). The estimation of the first derivative of the fitted trend of the model and its confidence interval allowed the identification of a transitional period when both bounded away from zero, starting after *ca*. 3000 cal years BP (Fig. 4b), in coincidence with the decline of herbivores (Fig. 3).

**Fig. 5:**
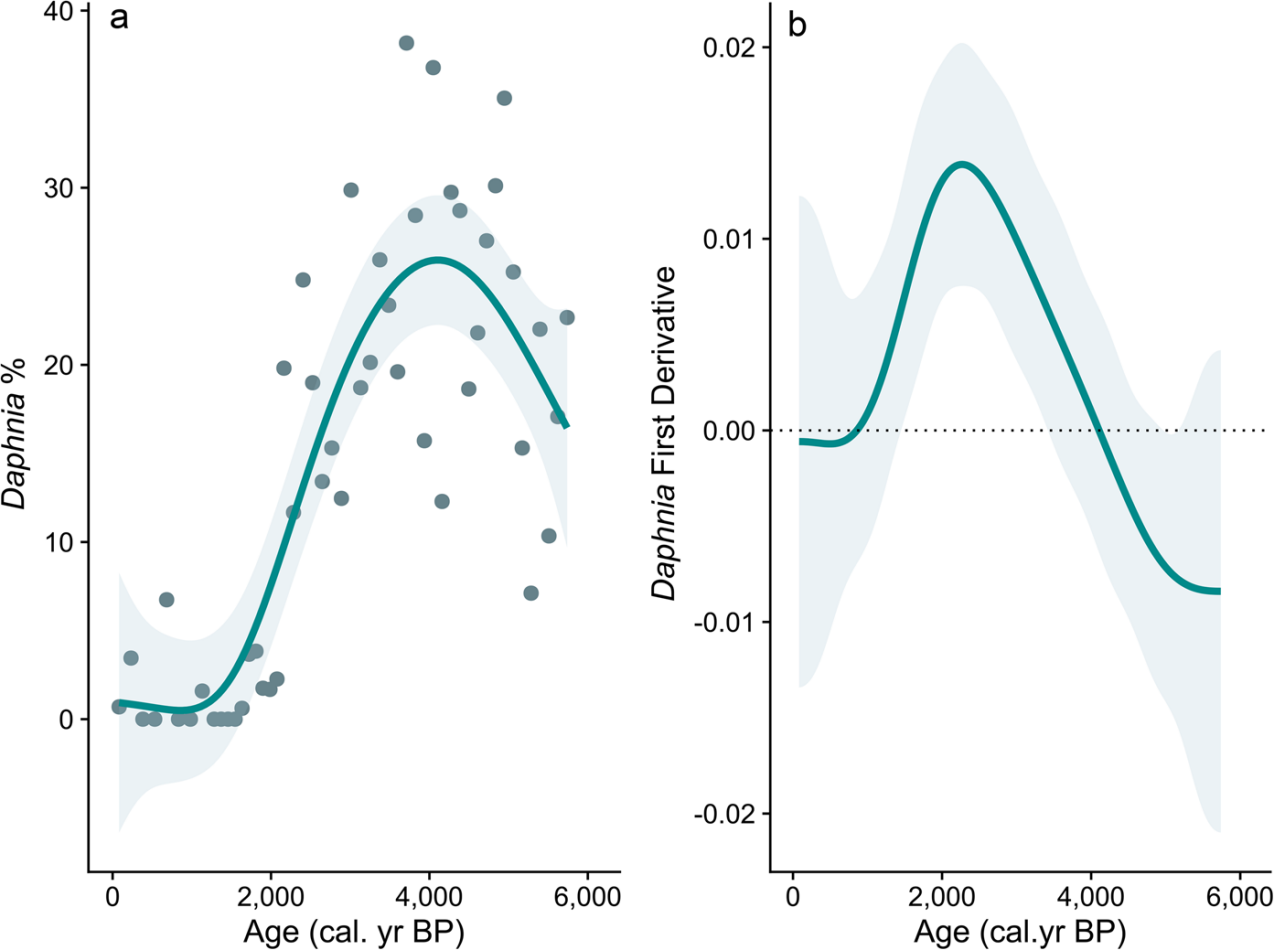
(**a**) Temporal contribution (%) of *Daphnia longispina* group in Diktar-Erik. GAM trend and 95% confidence interval fitted to the time series of *Daphnia*. (**b**) First derivative of the fitted GAM trend and its confidence interval, and the period of transition when both bounded away from the zero.

Considering temporal changes in the abundance of the keystone herbivore in the pelagic zone, we found that the *Daphnia longispina* group collapsed in coincidence with the transition from the Holocene Thermal Maximum to the subsequent cooling period, the Late Holocene (Figs. 2 and 5). The GAM fitted to the relative abundance of *Daphnia* across the time series captured 73.1% of the data deviance (*edf* of smooth term = 4.31, *P* value < 0.0001) (Fig. 5a). The estimation of the first derivative of the fitted trend and its confidence interval allowed the identification of a transition between *ca*. 3500 to 1500 cal years BP (Fig. 5b). This transition matches the timing of the beginning of the Late Holocene period (*ca*. 3500–500 cal years BP).

### In-lake and terrestrial drivers of compositional changes

**Fig. 6:**
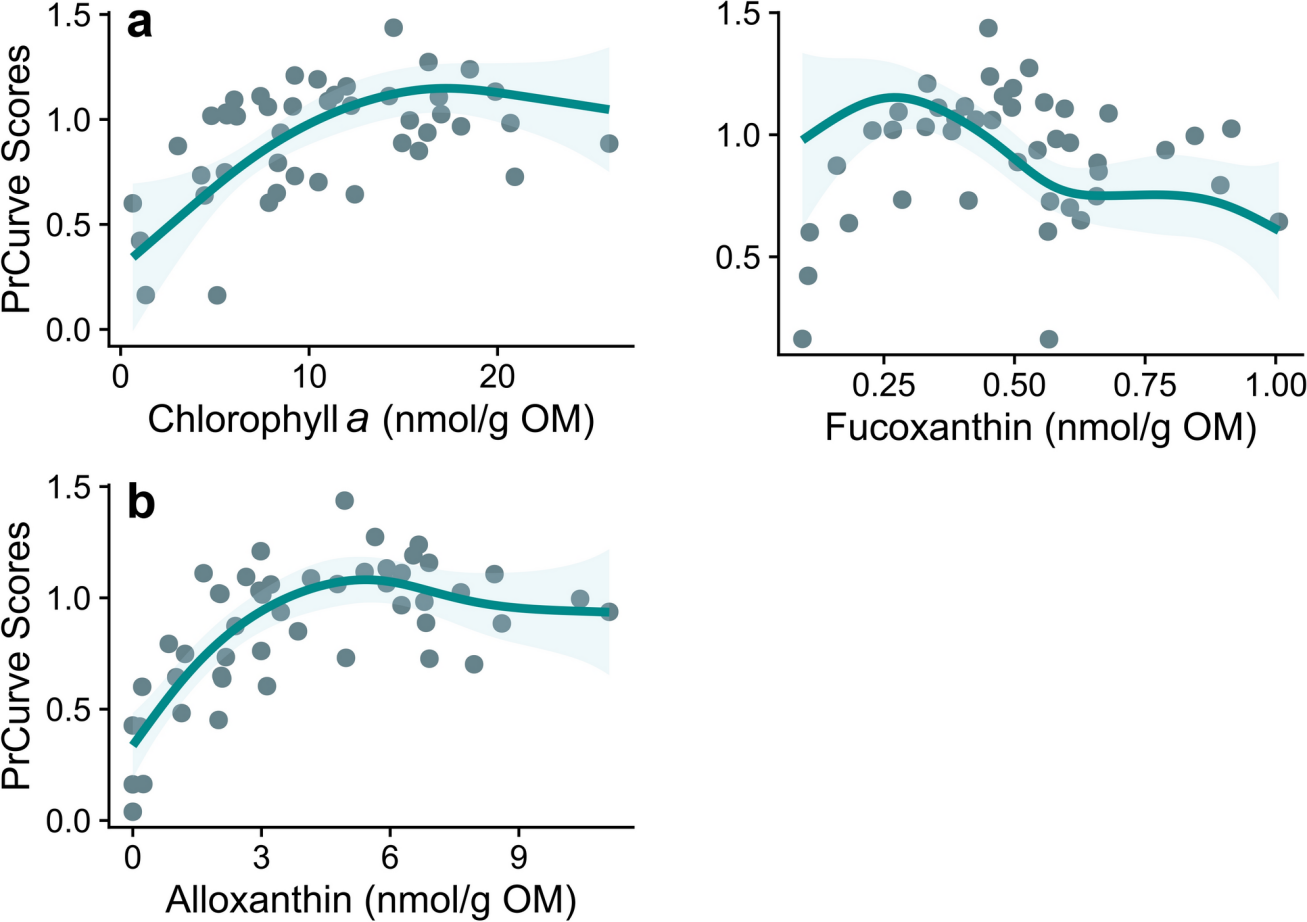
Selected GAM models showing the relationship between the scores of Principal curve analyses and distinct photosynthetic pigments determined in the same sediment core of Diktar-Erik^11^, (**a**) chlorophyll *a* and fucoxanthin (67.7% of explanation of the deviance) and (**b**) alloxanthin (62.3% of explanation of the deviance).

We modeled the relationship between compositional changes in Cladocera and pigment concentration estimated in the same sediment core^11^. Among the 15 GAM models linking Principal Curves to pigment concentrations, the model of chlorophyll *a* and fucoxanthin as explanatory variables and the one of alloxanthin show the strongest relationships with Principal Curve scores (Fig. 6; Supplementary, Table S2 online). These two models do not differ in more than two units in the AIC and explain a high percentage of the deviance (chlorophyll + fucoxanthin, 65.4%, and alloxanthin 62.3%, Supplementary Table S1 online). Principal Curve scores show a positive relationship with chlorophyll *a* (representing the biomass of primary producers) (*edf* = 2.8, *P* value < 0.0001) but negative with fucoxanthin, which is a proxy of diatoms, dinoflagellates, and chrysophytes (*edf* = 4.03, *P* value < 0.0001), whereas in another model, the scores and alloxanthin, which is a proxy of cryptophytes, display a positive trend (*efd* = 3.63, *P* value < 0.0001; Fig. 6).

**Fig. 7:**
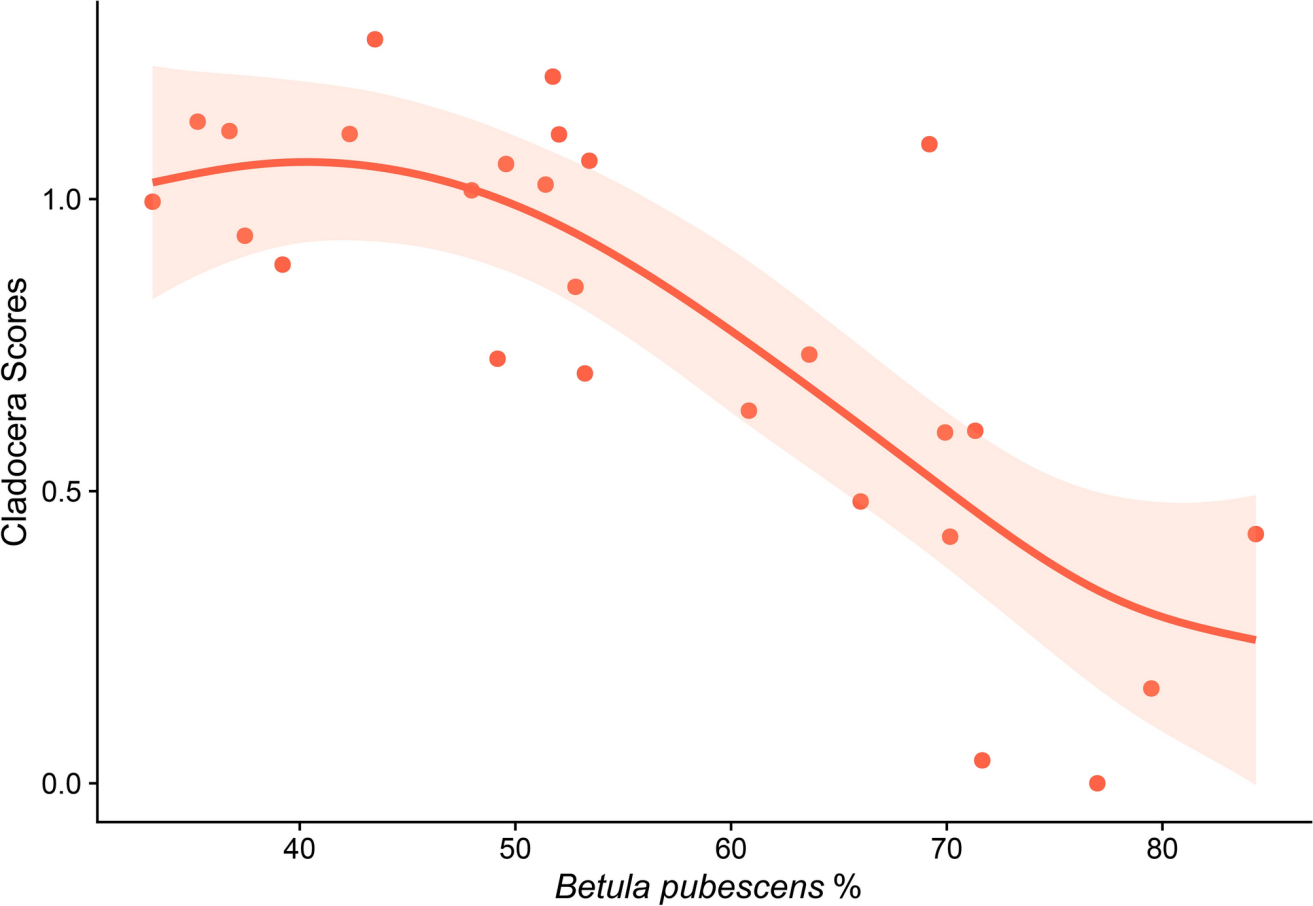
Fitted GAM trend and its 95% confidence interval for the relationship between Cladocera community Principal Curve scores in Diktar-Erik and the relative contribution (%) of *Betula pubescens* (mountain birch) reconstructed from the sediment core of Voulep Njakajaure in Abisko National Park^8^.

According to pollen reconstruction from Voulep Njakajaure in Abisko National Park^8^, the landscape transformed during the Holocene due to pine forest replacement with mountain birch (Supplementary, Fig. S4 online). This replacement occurred during the cooling period (*ca*. 3500–500 cal years BP), indicated by negative anomalies in temperature reconstructed for northern Europe^7^, which coincides with the decline in the Principal Curve scores for Cladocera and pigment concentration (Supplementary, Fig. S4 online). The fitted GAM indicates an inverse relationship between the cladoceran community and *Betula* contribution (*edf* of smooth term = 2.26, *P* value < 0.0001, 67.4% of deviance explanation; Fig. 7). The high contribution of *Betula* during the cooling period^8^ is associated with low scores of Principal Curves (Fig. 7), i.e., contracted cladoceran abundances, *Daphnia* and predators collapse, and a decline in taxonomic and horizontal diversity.

## Discussion

**Fig. 8:**
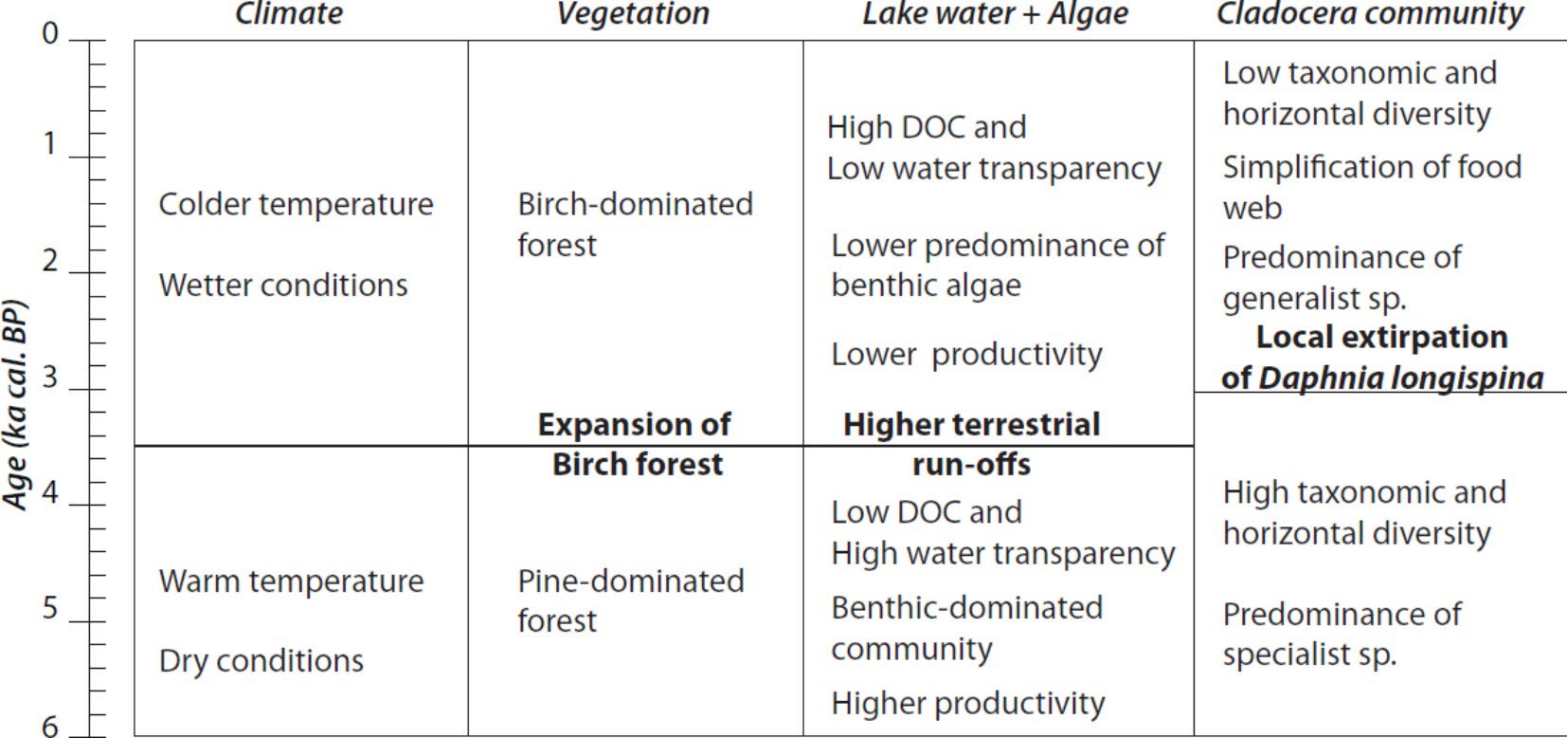
Conceptual summary of principal changes in climate, terrestrial vegetation, limnological characteristics of Diktar-Erik and Cladocera community during Mid and Late Holocene.

Our results represent the first record of Cladocera diversity over the last *ca*. 5700 years in a subarctic lake in Sweden and showed concordance with patterns of cladoceran diversity found in other paleolimnological studies in subarctic areas^18,19,29^. This study indicates that the Cladocera community in the subarctic lake Diktar-Erik underwent profound changes in composition over the last 5700 years, rendering a reduction of taxonomic and horizontal diversity during the Late Holocene (hypothesis 1). These changes were associated with a combination of aquatic and terrestrial processes driven by the long-term cooling^7,8^ observed from the end of the Holocene Thermal Maximum and during the Late Holocene (hypothesis 2) (Fig. 8).

The compositional changes in the cladoceran community associated with the transition from the Mid to Late Holocene can be characterized as a major decline in taxonomic and horizontal diversity and a restructuration of lake food webs, a process that started at *ca*. 3000 cal years BP (Fig. 8). This includes a reduction in the total number of predominant species from 18 to 7 which represents a reduction in horizontal diversity and trophic levels, due to the collapse of predators, and changes in ecological roles. During the Holocene Thermal Maximum, the lake had the highest species richness at different trophic levels, herbivores, and predators (horizontal diversity). For instance, in the littoral/benthic habitat, several species shared guilds, e.g., scrapers and generalist filter feeders, showing high trophic redundancy and distinct habitat preferences (phytophilous, and sandy bottom associated taxa). After the cooling in the Late Holocene, the food web in the lake started to change. This included a shift towards generalist and detritivore species (e.g., *C. sphaericus* group, *Acroperus elongatus, Leydigia* spp), causing a reduction of taxonomic and horizontal diversity (i.e., trophic redundancy) in the food web. In the pelagic habitat, the food web underwent a simplification, as it shifted from several herbivorous species and predators to the dominance of *Bosmina longispina*. Thus, the extirpation of the keystone herbivore *Daphnia*, and predators (*Leptodora kindtii*, *Bythotrephes longimanus and Polyphemus kindtii*) means a reduction of both horizontal diversity and trophic levels in the food web. Horizontal diversity increases the stability of the food web, i.e., the resistance and resilience due to perturbations^33,34^. As horizontal diversity and trophic levels are reduced with the climatic transition, it would be speculative to establish the effect on the food web stability. However, taxonomic diversity is positively associated with ecosystem functions^35^ and stability^34^. The reduction of species richness and the shifts of trophic guilds might lead to changes in trophic pathways and ecosystem functions such as biomass production and energy transfer when, for example, benthic phytophilous scrapers and filter feeders were replaced by detritivore or generalist species. Our findings, in line with earlier studies^18,29^, highlight that changes in cladoceran communities in arctic/subarctic lakes are driven by climate change, stressing that during warm periods specialist planktonic or macrophyte-associated species dominate the communities while during cold periods generalist species dominate (e.g., *C. sphaericus*, *A. elongatus*, *Alona* spp.). In addition to emphasizing this directional change and the loss of taxonomic diversity due to long-term cooling, our results also highlight the driving role of climate on another attribute of diversity, the horizontal dimension, and the structure of the food web.

The transition from the Holocene Thermal Maximum to the Late Holocene involved a shift from warm and dry conditions to an increase in moisture and a pronounced cooling by 1–2 °C compared with the time of the studies in the region^16,17^. Small temperature changes can lead to exponential changes in the metabolism and growth of ectotherm organisms (Metabolic Theory)^36^ such as cladocerans and algae. Indeed, changes in cladoceran composition, decline in abundances, and most species extirpation from the system occurred during the cooling phase as well as the decrease in the overall biomass of primary producers of the lake^11^. Total chlorophyll *a*, lutein, and alloxanthin, indicative of the total biomass of primary producers, chlorophytes, and cryptophytes^37^, respectively, showed the highest sediment pigment concentrations in the sediment record of Diktar-Erik during the Holocene Thermal Maximum, followed by a general decrease after 3500 cal years BP. Modeling the relation of Principal Curve scores of cladocerans, which summarized compositional changes, to photosynthetic pigments allowed to associate the decline in cladoceran abundances with the decline of primary producers (Fig. 8), and also, with a high-quality food resource, the cryptophytes. Thus, the low availability of food and/or high-quality food during the cooling period had a negative effect on specialist species such as *Camptocercus rectirostris* and *A. harpae* which are scrapers, and the keystone herbivore *Daphnia.* Moreover, the decline of *Daphnia* coincides with the beginning of the cooling period, showing an earlier response than the one detected for the whole cladoceran community. Several field and experimental studies demonstrate the depressing effect of low temperatures on *Daphnia* growth^38^ and the high demand (the double) for phosphorus-rich food in cold systems^39^ compared to temperate lakes^27^. The combination of lower temperatures, and fewer resources having a lower quality might have induced the collapse of specialist species, favoring generalist benthic littoral species or pelagic species like *Bosmina longispina* that feed efficiently under low food concentrations and in a selective mode, consuming highly nutritious flagellates^40,41^. In addition to temperature and food availability and quality as environmental factors influencing Cladocera diversity, fish predation may also have played a role. Arctic charr (*Salvelinus alpinus*, Linnaeus 1758) and brown trout (*Salmo trutta*, Linnaeus, 1758) are presently occurring in Dikar-Erik (see Methods for a description of the fish assemblage). Depending on species abundance and interspecific competition, the presence of trout induces Arctic charr to shift to pelagic and profundal resources whereas brown trout remain to exploit littoral resources^42^. In subarctic lakes, Arctic charr exhibits a seasonal shift towards consuming cladocerans in autumn and early winter, particularly when cladocerans are rich in lipids^26,42^. However, the lack of historical data on fish and their stocks makes it difficult to assess the significance of predation. If the impact of predation was at least moderate, we should expect a trophic cascade reflected in an increase in total chlorophyll *a* alongside the decline of *Daphnia* and the change in the size composition of zooplankton^43,44^. Instead, the observed pattern is the opposite: *Daphnia* have been extirpated, and chlorophyll *a* concentration has decreased (see Fig. 2, Supplementary Fig. S4). This suggests that temperature and food availability were the primary drivers of changes in Cladocera diversity. Fish predation may have influenced the cladoceran community or its size structure, probably to a lesser extent. Further studies are necessary to understand the combined effect of these environmental variables and their strength.

Numerous studies show how climate impacts on terrestrial and aquatic processes over time. For instance, warm and mild climatic conditions allowed the establishment of a pine forest (6300–4500 years cal BP) in northern Fennoscandia^17^ (Fig. 8). Climate cooling induced a decrease in terrestrial production and a gradual tree-line retreat in the studied area^8,16,17^, allowing the establishment of a mountain birch forest^8^ (Fig. 8). We found a negative relation between Cladoceran scores and the pollen contribution of mountain birch from a lake in the surrounding, indicating that compositional changes in cladocerans are not only linked to aquatic processes but also to landscape-driven processes. Regional increases in DOC in lakes occur during wetter climatic conditions and changes in terrestrial production and wetland cover^13,45,46^, linking terrestrial and lacustrine habitats^5^. During the Holocene, inferred total organic carbon and DOC increased in association with the deglaciation^47^ and during the cold and wet phase of the Late Holocene^9,10,47^. In addition, lakes in forested areas with connected mires, such as Diktar-Erik, showed an increase in total organic carbon during the period 4000–2500 BP due to changes in hydrology^47^ and also in coincidence with the development of the *Betula* forest. In Diktar-Erik, higher inputs of DOC and lower water transparency have previously been identified as a putative driver of changes in basal resource-sustaining *Daphnia* sp. biomass^11^.

The increase of DOC in lakes reduces light penetration and provides an important terrestrial input of organic matter, altering the relation between autotrophy and heterotrophy^5,13^. Reduction in light penetration also affects the thermal profile, decreasing both epilimnion depth, and hypolimnetic temperatures^14,48^, which in turn might impose physiological limitations on crustacean zooplankton diminishing its production^49^. The increased DOC levels constrain benthic primary production^13^, reducing food resources and the habitat structure for sediment- and macrophyte-associated species. In Diktar-Erik the chironomid community shifted to species indicative of high DOC conditions around 2000 cal years BP (e.g., *Heterotanytarsus*)^50^, which coincides with the decline of primary producers, the negative relationship between *Betula* contribution and cladocerans, and an increase in terrestrial carbon contribution to *Daphnia* as evidenced by ∂^13^C analyses of *Daphnia* ephippia^11^. Together, these pieces of evidence suggest the decline in air temperature and wetter conditions after 3500 cal years BP favored the delivery of DOC with impacts on the thermal structure and light penetration of Diktar-Erik, which operated as controlling variables of primary producers, zooplanktonic, and zoobenthic communities.

The response of lakes to climate change can vary depending on their size or location relative to the treeline^10^. Even when regional climate cooling began *ca*. 4000 cal years BP in Fennoscandia, lakes above the treeline responded more quickly than those in forested areas like Voulep Njakajaure^16^ or Diktar-Erik. For example for primary producer proxies, a shift in composition occurred at 4000–3500 cal years BP in tundra lakes^9,12^ while in forested lakes, like Voulep Njakajaure or Diktar-Erik, shifts occurred *ca*. 500–1000 years after the beginning of climatic cooling^11,16^. Cladocerans also shifted 1000 years later after the beginning of the climatic cooling^50^. These patterns highlight the importance of the catchment properties of a lake and its position relative to the ecotonal boundaries of the biota^17^ in the timing of the response to climatic drivers.

## Conclusion

In this study, we examined the past relationship between the cladoceran community and regional climate change over the last 5700 years in a subarctic lake. Our results support the hypotheses that climate cooling during the Late Holocene affected diversity attributes of the cladoceran community, reducing species richness, functional redundancy, and trophic levels. Our findings also indicate that changes in diversity were associated with a combination of aquatic and terrestrial processes driven by long-term cooling (Fig. 8). This highlights the close relationship between lake and terrestrial processes influenced by climatic controlling variables. Learning from past climate-induced changes in aquatic biodiversity may be used as an analog for future warming scenarios. Our study suggests that forecasted warming might promote a further shift in the cladoceran community characterized by an increased richness of specialist pelagic and benthic species and, thus, a greater complexity of benthic and pelagic pathways in subarctic lakes. However, the responses of lakes to global warming are complex as indicated by several studies showing inter-lake variability in their thermal and water clarity regimes and on lake depth^48,51^. The magnitude and impacts of Anthropocene warming on lakes and their aquatic communities may ultimately depend on how terrestrial ecosystems respond to warming, particularly regarding the feedback between terrestrial vegetation cover, DOC inputs, and lake-terrestrial interactions. Thus, responses are unlikely to be uniform. The rate of temperature change is much higher during the Anthropocene (approximately a 2 °C increase since the 1980s) compared to the observed during the Late Holocene (a 2 °C decrease over 3000 to 2000 years). Therefore, uncertainties exist on how this faster rate could produce imbalances and feedback between the terrestrial and the aquatic ecosystems, alleviating or potentiating warming effects on lakes and cladocerans, a pivotal link in food webs. Therefore, new research is needed to explore the interactions among lake depth (deep vs. shallow), DOC inputs, and varying rates of increased temperature to clarify the direction and speed of responses in aquatic communities to ongoing and future climatic changes in arctic and subarctic lakes.

## Methods

### Study area

Diktar-Erik´s lake (68°26′43″ N, 18°36′22″ E) is a small (0.1 km^2^) and relatively deep (16 m) oligotrophic (total phosphorus concentration *ca*. 7 µgP/L) lake located at 375 m.a.s.l. north of the Arctic Circle in Sweden (Fig. 1). The catchment area is 6.6 km^2^ and is currently covered by open land without vegetation (6%), open land with vegetation (59%), mountain birch forest (27%), wetlands (2%), and lakes and streams (7%)^52^. The catchment lies in the present-day ecotone between the mountain heath and mountain birch forest (Fig. 1) and was probably forested with pine forests during the Holocene Thermal Maximum, as the growth limit of pine was *ca.* 175 m higher in this region at that time^53^.

Arctic charr (*Salvelinus alpinus*) and brown trout (*Salmo trutta*) are generalist top predators. Both fish species are known to occur in the lake (pers. comm. Peter Ögren at the Association for Fish and Game Conservation in the municipality of Kiruna) and we are not aware of any records of historical fish assemblages in Diktar-Erik. However, Arctic charr, a cold tolerant and native fish, was probably the first pioneer fish to colonize freshwaters in Fennoscandia, followed by brown trout, after the end of the last glaciation around 13000 years BP^54^, while the ice in the study area retreated approximately 9500 years BP^55^. As the glacial rebound has not significantly altered watershed elevation, rivers remained accessible for long distances, making them suitable for the migration of these fish (Huifeldt-Kaas, 1923 in^54^). The waterfall and rapids downstream of Diktar-Erik create a natural barrier for other fish species that presently exist in the Torneträsk watershed, but not for Arctic charr or brown trout (pers. comm. P. Ögren and Prof. Göran Englund, Umeå University), as well as at its outlet where young-of-year and juveniles of brown trout occur^56^. Therefore, it is reasonable to conclude that both arctic charr and brown trout colonized the lake after the ice retreated.

Lakes are often used as sentinels and integrators of climate change. However, a major caveat is that anthropogenic changes, such as land use, can potentially mask or potentiate the effects of climate change^1,2^. Previous paleoecological studies from the area (Torneträsk watershed) have not identified distinguishable environmental impacts in response to early human activities, particularly those of the indigenous Sami people^57^. Historically, the Sami primarily engaged in hunting and fishing until the seventeenth century, and the effects of these activities are believed to have been minimal^58^. Between 1600 and 1900, there was a transition from hunting to intensive reindeer husbandry, which involved semi-nomadic pastoralism. Later, in the twentieth century, their main occupation shifted to extensive reindeer husbandry^59^. Palynological studies in northern Sweden confirmed a slight impact of these activities on local vegetation^58,60^. Thus, this minimal impact allows us to unravel how climate-induced changes during the Holocene affected Cladocera biodiversity.

### Sampling, age model, and data sources

In August 2017, we retrieved a 100 cm sediment core from the deepest part of the lake using a gravity corer (9 cm in diameter; UWITEC). We sliced the sediment core continuously from the top to 50 cm depth into 1 cm thick sub-samples. According to the age-depth model based on radiocarbon dates, the first 50 cm covers approximately 6000 years of the lake’s history, the limit between proglacial and lacustrine sediments^11^.

In this study, we present the data corresponding to Cladocera remains found in the sediment record of Diktar-Erik. To model the relationship between Cladocera and various environmental variables, we used multiple sources of information. We used photosynthetic pigment concentration analyzed in the same sediment record from Diktar-Erik^11^, pollen data of *Pinus* and *Betula pubescens*^8^ from a nearby lake (Vuolep Njakajaure) located in Abisko National Park, and the reconstructed temperature data from northern Europe^7^.

### Cladocera analyses

We prepared samples of Cladocera fossils from each stratigraphic level (every 1 cm) by heating wet sediment (6.86–12.18 g) in 10% KOH at 80 °C for 30–45 min. After that, we sieved the sample through a mesh of 45 µm under running tap water^61^. We took 200 µl subsamples to permanently mount on slides using glycerin and subsequently analyzed them under a light microscope (Zeiss Primo Star) at 200–400 × magnifications. We computed a minimum of 200 remains (carapaces, headshields, post-abdomens, claws, and ephippia) per sample, and then selected the most abundant body part to estimate species abundance^61^. For taxonomic identification, we used mainly an atlas^62^, and the names and authors follow the nomenclature in Dyntaxa—Swedish taxonomic database^63^. In addition, the association of species to specific habitats and trophic guild is detailed in Supplementary, Table S1 online.

### Statistical analyses

We identified a total of 26 different taxa, but almost 30% of them are considered rare due to low occurrence (e.g., found in only one layer), low numbers, or low contribution (see Supplementary Fig. S1). Because of this, they were not included in the statistical analyses. The statistical analyses presented below are based on the 18 most common taxa found throughout the core (see Fig. 2a).

The R 4.3.0 (2023-04-21) (R Core Team, 2023) provided the software for data analysis.

To perform stratigraphic diagrams we used the package *analogue*^64^.

To identify and summarize the changes in the composition of the Cladocera community, we performed a Principal Curves (PrC) analysis using the package *vegan*^65^. Principal curves (PrC) are a non- or semiparametric method suitable for indirect gradient analysis of multispecies abundance^66^. This methodology is better at describing changes in composition along a single dominant gradient, such as temporally ordered data than other ordination techniques such as PCA or CA^66^. First, we applied the Hellinger transformation, and after performing PrC analysis and extracting the scores, we modeled the times series using generalized additive models (GAMs). We choose this statistical tool because GAMs provide a superior alternative approach to trend estimation in paleoecological time series as can estimate non-linear trends, the magnitude of change, and the identification of periods of change, accounting for the lack of independence and providing statistical inference on each of these features^67^. To estimate the GAM we used the *gratia* package^68^, choosing the restricted maximum likelihood (REML) as the smoothness selection procedure. To account for the correlation between residuals, we performed a continuous-time first-order autoregressive process, CAR(1)^67^. Finally, to identify periods of transition, we estimated the first derivative of the fitted trend and its confidence intervals. We identified transitions as those periods where the first derivative of the fitted trend and its interval bounded away from zero^67^. To find out if the species replacement or collapse matches the timing of the climatic transition, *ca*. 3500 cal years BP, we selected an emblematic species, *Daphnia longispina* group, based on its importance as a keystone grazer, nutrient recycler, and food quality requirements^39^. We applied GAM to its temporal series following the procedures described above.

To estimate changes in taxonomic diversity along the time series we estimated Shannon’s diversity index (H’)^69^, and was calculated as

$${H}{\prime}=-\sum_{i=1}^{R}pi lnpi$$

where *p*_*i*_ is the proportion of the *i*th taxon in each sample. To estimate changes in horizontal diversity we estimated species richness at two trophic levels: primary consumers (all pelagic, littoral, and benthic herbivores) and secondary consumers (pelagic and littoral predators). We modeled the temporal trends of diversity (Shannon index and horizontal diversity) by applying GAMs and detected transition following the above-mentioned procedures^67^.

To identify potential drivers of processes within the lake associated with compositional changes in the cladoceran community, we adjusted GAM models to the scores of the Principal Curve analysis versus photosynthetic pigments as proxies of lake production or functional groups. In particular, we used chlorophyll *a*, alloxanthin, fucoxanthin, and diatoxanthin concentrations published^11^ as explanatory variables. In all cases, we used the Gaussian family to fit the models, except for the model with chlorophyll *a* and fucoxanthin as explanatory variables, where we used the scaled t family (Fig. 6a).

To understand the link with terrestrial processes that could affect the cladoceran community, we considered the shift in the forest composition from pine to mountain birch induced by climate cooling from a nearby area in Abisko National Park^8,16^. To do so, we adjusted a GAM to the scores of the Principal Curve analysis versus the relative abundance of mountain birch. We used the reconstruction of the birch contribution^8^ for the analysis. We matched the values of explanatory and response variables considering the reconstructed chronologies of the data.

## Supplementary Information

Supplementary Information.

## Data Availability

Data (cladoceran contribution and Shannon index from the sediment core of Diktar-Erik) will be available upon reasonable request. Please contact M. Ángeles González Sagrario at gonsagra@gmail.com.
