# Supplementary material for: Late Holocene cooling drove drastic decreases in cladoceran diversity in a subarctic lake
Source: Sci Rep. 2024 Dec 16;14:30490. doi: 10.1038/s41598-024-81690-7 (PMC11649896; doi:10.1038/s41598-024-81690-7)
Supplement: Supplementary file 1 — Supplementary Information. [file 41598_2024_81690_MOESM1_ESM.docx]

**Supplementary information.**

**Late Holocene cooling drove drastic decreases in cladoceran diversity in a subarctic lake.**

**María de los Ángeles González Sagrario^1^, Tobias Vrede^2^, and Simon Belle***^2^

^1^: Instituto de Investigaciones Marinas y Costeras (IIMYC), Facultad de Ciencias Exactas y Naturales, Universidad Nacional de Mar del Plata, CONICET, J. B. Justo 2550, (7600) Mar del Plata, Argentina

^2^: Swedish University of Agricultural Sciences**,** Department of Aquatic Sciences and Assessment, Box 7050, 750 07 UPPSALA, Sweden

* Corresponding author: Simon Belle, simon.belle@slu.se

**Data set selection**

For the data selection, we considered the number of samples where the taxa occurred, the number of remains, and its relative abundance. We identified a total of 26 taxa, of which 18 taxa occurred in at least 8 of the 50 samples of the sediment core, contributing with a minimum of 28 and a maximum of 8481 remains. These common taxa are depicted in black in Fig. S1. In addition, we also found 8 rare taxa that appeared sporadically in only 1 to 4 samples and with a contribution of 2 to 15 remains in total, representing low abundances (usually less than 2 %, Fig. S1, taxa in red). These rare taxa were *Camptocercus* sp., *Alona intermedia*, *Alona costata*, *Pleroxus* sp., *Monospilus*, *Alonella exigua*, *Disparalona* sp. and *Graptoleberis testudinella*.


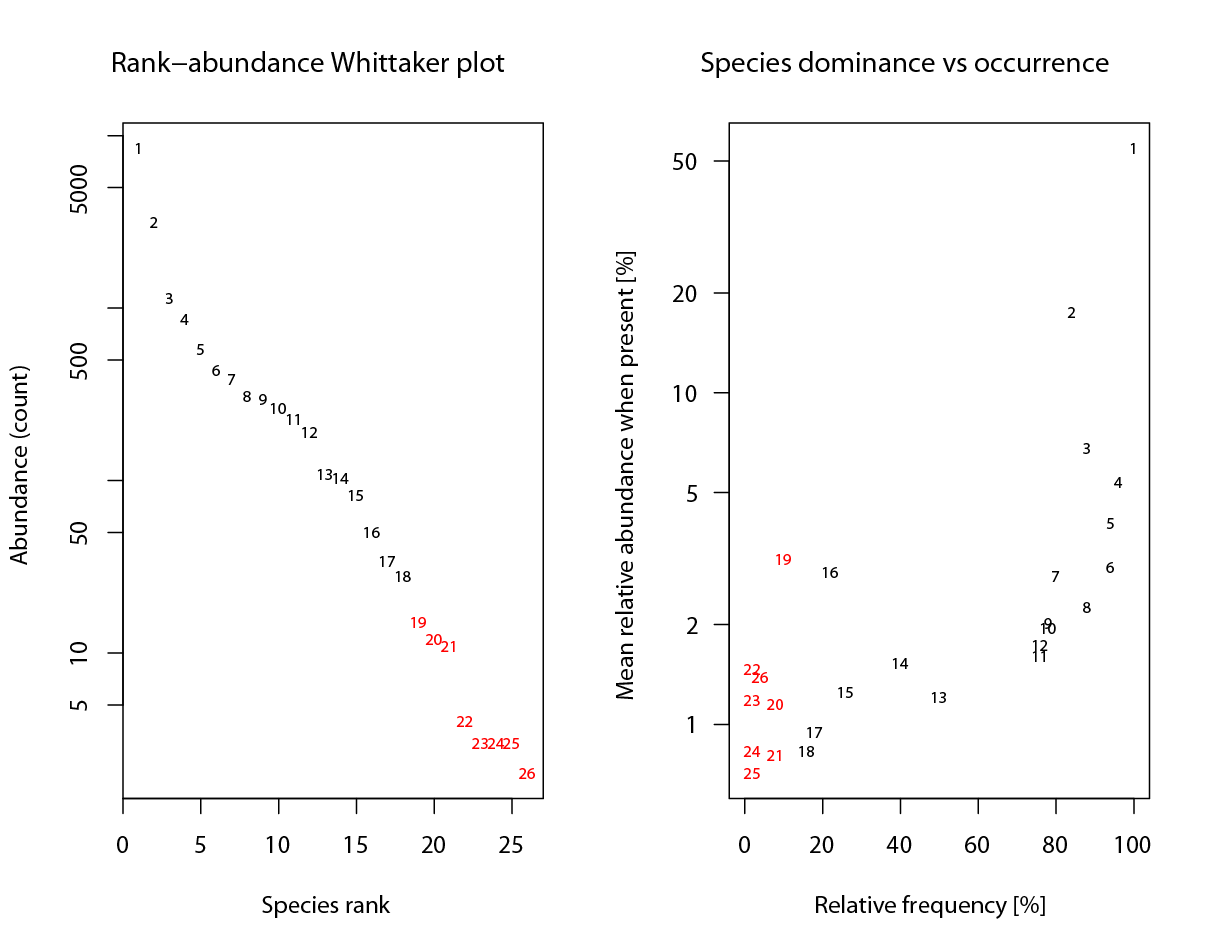


**Figure S1**. Whittaker Plot showing abundance (number of remains) and species rank (left panel) and the relationship between taxa dominance and occurrence in the sediment core of Lake Diktar Erik. Taxa identification: 1: *Bosmina longispina*, 2: *Daphnia longispina* group, 3: *Alonella nana*, 4: *Acroperus harpae*, 5: *Chydorus sphaericus* group, 6: *Alona rustica*, 7: *Leptodora kindtii*, 8: *Acroperus elongatus*, 9: *Ophryoxus gracilis*, 10: *Paralona pigra*, 11: *Alona affinis*, 12: *Eurycercus* spp., 13: *Camptocercus rectirostris*, 14: *Alonella excisa*, 15: *Bythotrephes longimanus*, 16: *Leydigia* spp., 17: *Oxyurella tenuicaudis*, 18: *Polyphemus pediculus*, 19: *Camptocercus* sp., 20: *Alona intermedia*, 21: *Pleuroxus* spp., 22: *Monospilus* sp., 23: *Alona costata*, 24: *Alonella exigua*, 25: *Disparalona* sp., 26: *Graptoleberis testunidaria*.

We selected the 18 common taxa to run all the analyses presented in this study because predominant taxa are the most influential in driving diversity patterns. For example, we compared the estimation of the Shannon Index using the entire dataset (26 taxa) and only the most common species/genera (18 taxa), and found a high overlap of the values (Fig. S2), and, in addition, the same diversity trend. Diversity showed an increasing trend during the Holocene Thermal Maximum and a decline during the Late Holocene for both sets of data (Fig. S2).

**
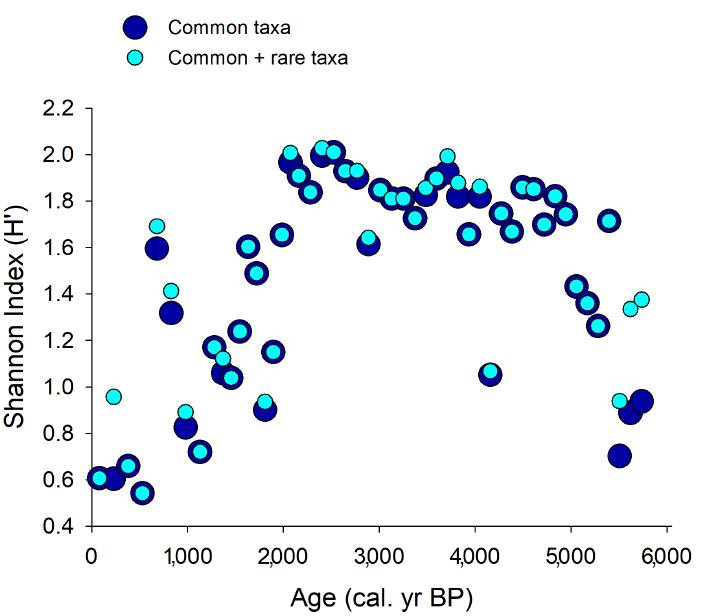
**

**Figure S2**. Comparison of Shannon index considering the whole data set, common plus rare taxa (26), and only common taxa (18).

**Trophic guilds and preferred habitat of cladoceran species**.

We assigned habitat preferences and trophic guilds to each taxon according to published references (Table S1).

**Table S1**. Trophic guilds and habitat preferences of cladoceran taxa identified in the sediment record of Diktar-Erik´s lake. References: [^[1]^](https://www.zotero.org/google-docs/?IiAlqw)*Bosmina longispina*, *Daphnia longispina* group; [^[2]^](https://www.zotero.org/google-docs/?PPQXes)*Leptodora kindtii;* [^[3]^](https://www.zotero.org/google-docs/?y1Xsud)*Bythotrephes longimanus;* [^[4]^](https://www.zotero.org/google-docs/?qM0JRX)*Polyphemus pediculus;* [^[5]^](https://www.zotero.org/google-docs/?yCmepJ)*Acroperus elongatus, Acroperus harpae, Camptocercus rectirostris*, *Eurycercus* spp.*;* [^[6]^](https://www.zotero.org/google-docs/?DMbi5g)*Alonella nana, Alonella excisa, Chydorus sphaericus, Paralona pigra, Leydigia* spp*., Alona affinis;* [^[7]^](https://www.zotero.org/google-docs/?4g8RCh)*Alona rustica, Oxyurella tenuicaudis, Ophryoxus gracilis.*

| **Taxon** | **Habitat** | **Trophic guild** |
| --- | --- | --- |
| *Bosmina longispina* | pelagic | filter feeder, herbivore |
| *Daphnia longispina* | pelagic | filter feeder, herbivore |
| *Leptodora kindtii* | pelagic | predator |
| *Bythotrephes longimanus* | pelagic | predator |
| *Polyphemus pediculus* | littoral | predator |
| *Acroperus elongatus* | littoral/benthic, in vegetated areas /sandy bottom | generalist, scraper, herbivore |
| *Acroperus harpae* | littoral /sandy bottom | scraper, herbivore |
| *Camptocercus rectirostris* | littoral in vegetated areas | scraper, herbivore |
| *Eurycercus* spp | littoral in vegetated areas | scraper, herbivore |
| *Alonella nana* | littoral/benthic, in vegetated areas | scraper, herbivore |
| *Alonella excisa* | littoral/benthic, in vegetated areas | scraper, herbivore |
| *Ophryoxus gracilis* | littoral/benthic, in vegetated areas/sandy bottom | scraper/filter feeder, herbivore |
| *Oxyurella tenuicaudis* | littoral, in vegetated areas | scraper, herbivore |
| *Chydorus sphaericus* | littoral | generalist, scraper/filter feeder, herbivore |
| *Alona rustica* | littoral/benthic | generalist, scraper, herbivore |
| *Alona affinis* | littoral/benthic | generalist, scraper/filter feeder, herbivore |
| *Leydigia* spp. | benthic | detritivore |
| *Paralona pigra* | benthic | detritivore |

**Temporal changes in taxonomic diversity**.

To detect transitions on the temporal trend of taxonomic diversity we adjusted a GAM[^[8]^](https://www.zotero.org/google-docs/?WQiMC0) to the temporal series of the Shannon Index following the procedures[^[9]^](https://www.zotero.org/google-docs/?BWtKDd) described in the main text. The GAM fitted to the temporal series of the Shannon diversity index explains 67.7% of the variation (e*df* of smooth term=4.05, *p*-value < 0.0001). The first derivative identifies two distinct periods of change (Fig. S3). From *ca*. 5700 to 2400 cal. yr BP, Shannon’s diversity index shows an increasing trend, while after 2,400 cal yr. BP diversity decreased.


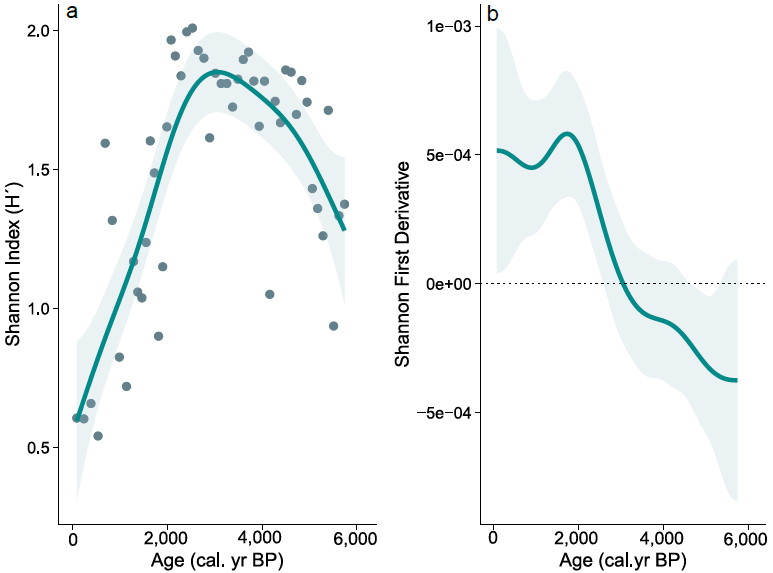


**Figure S3**. Temporal trend of the Shannon Index. **a** GAM trend with a 95% confidence interval fitted to the temporal series of species richness (H´).**b** The first derivative of the fitted GAM trend and its confidence interval. The transition period corresponds to those time points where the trend and its interval are away from zero.

**Relationship between compositional changes in Cladocera and primary producers**

To understand in-lake processes that could have driven the changes in the cladoceran community along the time series, we adjusted GAMs to the scores of the Principal Curve analysis versus photosynthetic pigments as proxies of lake production or functional groups. Scores of the Principal Curve analysis reflect compositional changes in the cladoceran community. In particular, we used chlorophyll a, alloxanthin, fucoxanthin, and diatoxanthin concentrations analyzed in the same sedimentary core[^[10]^](https://www.zotero.org/google-docs/?6651qm) as explanatory variables.

Table S2 summarizes all the models explored (15 in total) and the selected models based on the percentage of deviance explained, the AIC, and the qualitative evaluation of the diagnostics of each model. The results indicate that the model of chlorophyll and fucoxanthin as explanatory variables and the one of alloxanthin show a strong relationship with Principal curve scores, i.e., with compositional changes. These models do not differ in more than two units in the AIC and explain a high percentage of the deviance (chlorophyll + fucoxanthin, 65.4%, and alloxanthin 62.3%, Table S1, Fig. 6 in main text). Principal Curve scores show a positive relationship with chlorophyll *a* (representing the overall primary lake production) but negative with fucoxanthin (proxy of diatoms, dinoflagellates, and chrysophytes), whereas a positive relation with alloxanthin (proxy of cryptophytes).

| **Table S2**. Statistics after fitting Generalized Additive models to the scores of Principal Curves (PrC) and photosynthetic pigment proxies as explanatory variables. Statistics include the estimated degrees of freedom (*edf*) (the order corresponds with the variable order in the model), *P-values* of each smooth term, the percentage (%) of the deviance explained by the fitted model, and Akaike Criteria (AIC). We estimated all fitted models using the Gaussian distribution and REML smoothness selection and set the significance level on *P-values* < 0.05. Models in blue represent the best-fitted models according to AIC, percent of deviance explained, and qualitative evaluation of diagnostics of the model. Symbols "*", "**" and "***" denote *P*-values< 0.01, 0.001, and 0.0001, respectively. Marginally significant values are shown; non-significant values are referred to as "ns". Acronyms: Chla: Chlorophyll *a*, Allo: Alloxanthin, Fuco: Fucoxanthin, Diato: Diatoxanthin. | | | | |
| --- | --- | --- | --- | --- |
| **Model** | ***edf* of smooth** | ***P*-value** | **% deviance** | **AIC** |
| PrC ~s(Chla) + s(Allo)+ s(Fuco)+ s(Diato) | 2.62 | ns | 69.8 | -15.06 |
|  | 3.15 | *** |  |  |
|  | 1 | 0.06 |  |  |
|  | 1 | ns |  |  |
| PrC ~s(Chla) + s(Allo)+ s(Diato) | 1.7 | ns | 65.4 | -1.76 |
|  | 3.15 | *** |  |  |
|  | 1 | ns |  |  |
| PrC ~s(Chla) + s(Allo)+ s(Fuco) | 2.7 | ns | 70.1 | -17.1 |
|  | 3.33 | *** |  |  |
|  | 1 | ns |  |  |
| PrC ~s(Chla) + s(Diato)+ s(Fuco) | 3.89 | *** | 67.7 | -13.09 |
|  | 1 | ns |  |  |
|  | 2.21 | *** |  |  |
| **PrC ~s(Chla) + s(Fuco)** | **2.8** | ******* | **65.4** | **-14.48** |
|  | **4.03** | ******* |  |  |
| PrC ~s(Chla) + s(Allo) | 1.52 | ns | 64.6 | -13.14 |
|  | 3.56 | *** |  |  |
| PrC ~s(Chla) + s(Diato) | 3.07 | *** | 41.9 | 7.47 |
|  | 1 | ns |  |  |
| PrC ~ s(Allo)+ s(Fuco)+ s(Diato) | 3.53 | *** | 65.5 | -14.46 |
|  | 1 | ns |  |  |
|  | 1 | ns |  |  |
| PrC~ s(Allo)+ s(Fuco) | 3.9 | *** | 65.8 | -16.37 |
|  | 1 | ns |  |  |
| PrC ~ s(Allo)+ s(Diato) | 3.26 | *** | 63.8 | -13.7 |
|  | 1.23 | ns |  |  |
| PrC ~ s(Fuco)+ s(Diato) | 5.39 | *** | 58.4 | -3.71 |
|  | 1 | 0.08 |  |  |
| PrC ~ s(Chla) | 3.1 | *** | 41.9 | 5.52 |
| **PrC ~ s(Allo)** | **3.62** | ******* | **62.3** | **-14.56** |
| PrC ~ s(Diato) | 3.98 | * | 26.7 | 26.7 |
| PrC ~ s(Fuco) | 5.3 | *** | 54.9 | -1.74 |
|  |  |  |  |  |

**Trends for pigments and Cladocera in Diktar-Erik, temperature anomalies reconstructed for Northern Europe, and pollen trends from birch and pine.**

According to pollen reconstruction from Voulep Njakajaure in Abisko National Park[^[8]^](https://www.zotero.org/google-docs/?fSlMRO), the landscape transformed during the Holocene due to pine forest replacement with mountain birch (Fig. S4 online). This replacement occurred during the cooling period (*ca*. 3,500 – 500 cal. yr BP), indicated by negative anomalies in temperature reconstructed for northern Europe[^[7]^](https://www.zotero.org/google-docs/?5aKR64), which coincides with the decline in the Principal Curve scores for Cladocera and pigment concentration (Fig. S4 online).


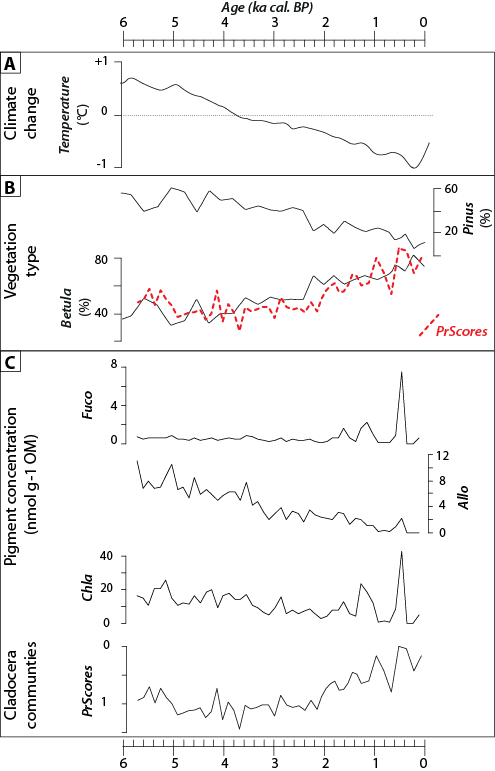


**Figure S4.** Temporal trends of temperature anomalies for Northern Europe[^[11]^](https://www.zotero.org/google-docs/?RoPPEW) inferred from pollen analyses (A), pollen from mountain birch (*Betula)* and pine (*Pinus)* in a sediment core from Voulep Njakajaure in Abisko National Park[^[12]^](https://www.zotero.org/google-docs/?xKJsXD) and principal Curve scores of the Cladocera community (red broken line) (B), and pigment concentration of the sediment record of Lake Diktar-Erik (C).

**References**.

[1. DeMott, W. R. Feeding selectivities and relative ingestion rates of Daphnia and Bosmina. *Limnol. Oceanogr.* **27**, 518–527 (1982).](https://www.zotero.org/google-docs/?M0ZyVI)

[2. McNaught, A. S., Kiesling, R. L. & Ghadouani, A. Changes to zooplankton community structure following colonization of a small lake by Leptodora kindti. *Limnol. Oceanogr.* **49**, 1239–1249 (2004).](https://www.zotero.org/google-docs/?M0ZyVI)

[3. Pothoven, S. A. *et al.* Influences on *Bythotrephes longimanus* life-history characteristics in the Great Lakes. *J. Gt. Lakes Res.* **38**, 134–141 (2012).](https://www.zotero.org/google-docs/?M0ZyVI)

[4. Xu, L. *et al.* Biogeography and evolution of the Holarctic zooplankton genus Leptodora (Crustacea: Branchiopoda: Haplopoda). *J. Biogeogr.* **38**, 359–370 (2011).](https://www.zotero.org/google-docs/?M0ZyVI)

[5. Duigan, C. A. The ecology and distribution of the littoral freshwater Chydoridae (Branchiopoda, Anomopoda) of Ireland, with taxonomic comments on some species. *Hydrobiologia* vol. 241 1–70 (1992).](https://www.zotero.org/google-docs/?M0ZyVI)

[6. Fryer, G. Evolution and Adaptive Radiation in the Chydoridae (Crustacea: Cladocera): A Study in Comparative Functional Morphology and Ecology. *Philos. Trans. R. Soc. Lond. B. Biol. Sci.* **254**, 221–385 (1968).](https://www.zotero.org/google-docs/?M0ZyVI)

[7. Błędzki, L. A. & Rybak, J. I. *Freshwater Crustacean Zooplankton of Europe*. (Springer, 2016).](https://www.zotero.org/google-docs/?M0ZyVI)

[8. Wood, S. N. *Generalized Additive Models: An Introduction with R*. (Chapman and Hall/CRC, 2017).](https://www.zotero.org/google-docs/?M0ZyVI)

[9. Simpson, G. L. Modelling palaeoecological time series using generalised additive models. *Front Ecol Evol* **6**, 149 (2018).](https://www.zotero.org/google-docs/?M0ZyVI)

[10.Belle, S. *et al.* Climate-induced changes in carbon flows across the plant-consumer interface in a small subarctic lake. *Sci. Rep.* **9**, 17087 (2019).](https://www.zotero.org/google-docs/?M0ZyVI)

[11.Seppä, H., Bjune, A. E., Telford, R. J., Birks, H. J. B. & Veski, S. Last nine-thousand years of temperature variability in Northern Europe. *Clim Past* **5**, 523–535 (2009).](https://www.zotero.org/google-docs/?M0ZyVI)

[12.Barnekow, L. Holocene regional and local vegetation history and lake-level changes in the Torneträsk area, northern Sweden. *J. Paleolimnol.* **23**, 399–420 (2000).](https://www.zotero.org/google-docs/?M0ZyVI)
